# "K" EY rules to improve learning skills in forth arm robotic platform

**DOI:** 10.1590/0102-67202026000001e1930

**Published:** 2026-05-18

**Authors:** André Vicente BIGOLIN, Leandro Totti CAVAZZOLA, Antonio Nocchi KALIL

**Affiliations:** 1 Irmandade Santa Casa de Porto Alegre, Robotic Surgery Service and Robotic Surgery Training Center – Porto Alegre (RS), Brazil.; 2 Universidade Federal do Rio Grande do Sul, Hospital de Clínicas de Porto Alegre, General Surgery Service – Porto Alegre (RS), Brazil.; 3 Irmandade Santa Casa de Porto Alegre, Hospital Santa Rita, Technical Directorate – Porto Alegre (RS), Brazil.; 4 Irmandade Santa Casa de Porto Alegre, Research Directorate – Porto Alegre (RS), Brazil.; 5 Universidade Federal de Ciências da Saúde de Porto Alegre, Departament of Surgery – Porto Alegre (RS), Brazil.

**Keywords:** Robotic Surgical Procedures, Professional Training, High Fidelity Simulation Training, Procedimentos Cirúrgicos Robóticos, Capacitação Profissional, Treinamento com Simulação de Alta Fidelidade

## Abstract

**Background:**

The number of robotic surgeries in the world grows every year. There is pressure to train surgeons, who face the challenge of transforming their surgical technique and adapting to new technology. However, there is a gap in training standardization that allows improvements to be assessed. One of the main technical challenges of the robotic platform with four working arms is their simultaneous cooperation through the movement itself. The technique presented in this article was developed based on the training of more than 400 robotic surgeons.

**Aims:**

To describe the steps to be carried out when performing the training: activation of the arm during "swap" maneuvers (changing active instrument) during one surgical procedure, to understand the movement of the 4th robotic arm, using the letter "K" as a reference.

**Technique:**

The screen will be divided into right and left and, then, the side of the screen that houses two working arms will be divided into three quadrants — top, bottom and middle. There are four basic rules that will mitigate the learning curve, avoiding the main mistakes that lead to considerations of instruments and difficulty in intracavitary mobility.

**Conclusions:**

The K technique is a relevant resource in teaching robotic surgery, offering a simple and reproducible methodology for developing important skills to reduce the learning curve for new robotic surgeries.

## ARTICLE HIGHLIGHTS

The "K" technique provides a simple and reproducible method to improve coordination and control of the four robotic arms.

Developed from the training of more than 400 robotic surgeons, it helps reduce the learning curve in robotic surgery.

The technique enhances essential robotic skills such as depth perception, bimanual dexterity, and instrument control.

It serves as a practical tool for both dry-lab and virtual reality training environments.

## Summary

**CENTRAL MESSAGE:**

The use of robotics has emerged as a major innovation in the area of minimally invasive surgery in recent decades. Despite technology, results still depend on the technical capacity of professionals. Working with three-dimensional images, using immersive technology and having total control of robotic arm commands by the surgeon are the biggest paradigm shifts that the robot offers when compared to laparoscopic surgery. This technical note, using the letter "K" as a reference, was created with the experience of professionals involved in the training of more than 400 surgeons from different specialties. The aims is to build an easily reproducible mnemonic method to facilitate the adaptation of surgeons to the synchronized work of a 4-arm robotic platform.

**PERSPECTIVES:**

The "K" technique is a relevant technical resource in the teaching of robotic surgery, as it provides the opportunity for the development of important skills for safety and optimization of surgical time. It is easily reproducible and can serve as a basis for building virtual reality training models that explore its concepts at an even more affordable cost for a large scale of training.

## VISUAL ABSTRACT

**Figure:**
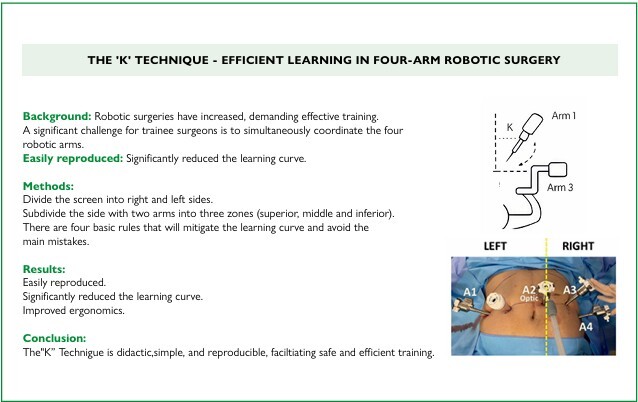

## RESUMO

**Racional:**

O número de cirurgias robóticas no mundo cresce a cada ano. Há uma pressão para treinar cirurgiões, que enfrentam o desafio de transformar sua técnica cirúrgica e se adaptar às novas tecnologias. No entanto, há uma lacuna na padronização do treinamento que permita avaliar melhorias. Um dos principais desafios técnicos da plataforma robótica com quatro braços de trabalho é a cooperação simultânea deles por meio do próprio movimento. A técnica apresentada neste artigo foi desenvolvida com base no treinamento de mais de 400 cirurgiões robóticos.

**Objetivos:**

Descrever os passos a serem seguidos durante o treinamento: ativação do braço robótico durante manobras de troca (alteração do instrumento ativo) em um procedimento cirúrgico, compreendendo o movimento do 4º braço robótico, utilizando a letra "K" como referência.

**Técnica:**

A tela será dividida em direita e esquerda e, em seguida, a lateral da tela que abriga dois braços de trabalho será dividida em três quadrantes - superior, inferior e central. Existem quatro regras básicas que atenuarão a curva de aprendizado, evitando os principais erros que levam a considerações de instrumentos e dificuldade na mobilidade intracavitária.

**Conclusões:**

A técnica K é um recurso relevante no ensino de cirurgia robótica, oferecendo uma metodologia simples e reprodutível para o desenvolvimento de habilidades importantes para reduzir a curva de aprendizado de novas cirurgias robóticas.

## INTRODUCTION

 The use of robotics has emerged as a major innovation in the area of minimally invasive surgery in recent decades. Its diffusion has gained exponential representation in recent years. The da Vinci model is the most widespread in the world, with over 7,500 platforms installed worldwide until 2022^6,11^. 

 Most platforms developed for cavitary surgeries do not have autonomous features. The great attraction of this technique is the expansion of the technical resources that the surgeon can have to perform the procedures. Despite technology, results still depend on the technical capacity of professionals. Working with three-dimensional images, using immersive technology and having total control of robotic arm commands by the surgeon are the biggest paradigm shifts that the robot offers when compared to laparoscopic surgery. This can also be a major challenge for teaching this technique. It is clear that there is a learning curve, which appears to be more challenging in surgeons less exposed to technology and minimally invasive tools^2,3,8,12^. 

 Therefore, there is a need to develop standardized training strategies that facilitate the acquisition of knowledge and expand the use of technology resources, increasing surgeons’ adherence and softening learning curves^4,7^. 

 This technical note was created with the experience of professionals involved in the training of more than 400 surgeons from different specialties and using protocols that respected the regulations of the Brazilian Medical Association and the Brazilian College of Surgeons^1^. It aims to build an easily reproducible mnemonic method to facilitate the adaptation of surgeons to the synchronized work of a 4-arm robotic platform. 

 We have observed that one of the greatest difficulties for surgeons’ training in robotic surgery is finding the appropriate working proximity and simultaneously coordinating camera control and control of other arms dynamically and without collisions^9^. Some skills can be trained in virtual reality (VR)^10,13-16^. 

 However, practical training is essential. Raison et al. showed that dry-lab training offers significantly greater improvements than VR simulation^13,15^. We identified that dry-lab training with the platform was able to increase the average knowledge acquisition on the platform by x% when applied to students with prior web-based training. Among the activities of this training, the one that was recognized by the students as most effective was the technique recommended by the main author (A.V.B.), which consists in understanding the movement of the fourth robotic arm using the letter "K" as a reference to describe the steps to be followed during arm activation in "swap" maneuvers (changing the active instrument) during the procedure. 

## METHODS

### Description of the technique

**Figure 1:**
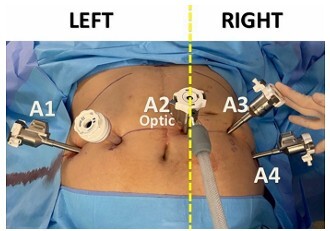
Positioning of the four robotic arms represented by A1, A2, A3 and A4. To the right of the position, marked for use of the vision system (A2, optics), two arms (A3 and A4) are positioned which will be operated with the surgeon’s right hand on the console.

 The planning for coupling the robotic arms of the da Vinci 4^th^ and 5^th^ generation platform provides for the use of up to four arms to carry out the procedures. One of them is dedicated to placing the optics. The number of arms on each side of this position determines which of the surgeon’s hands will command one or two forceps (Figure 1). 

 The surgical field vision area is divided into two fields, the right representing the working area of the arm controlled by the surgeon’s right hand, and the left representing the work area controlled by the surgeon’s left hand. 

**Figure 2:**
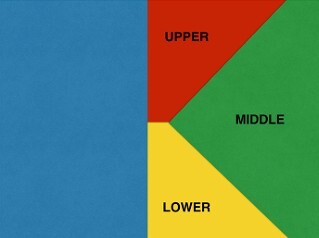
The side where two arms will work will be divided into three work zones: top, side and bottom. This forms a letter K when this is the right side and the letter K inverted when it is the left side.

 The side where two arms will work will be divided into three work zones: top, side and bottom (upper, middle and lower). This forms a letter K when on the right side, and an inverted K when on the left side (Figure 2). 

**Figure 3:**
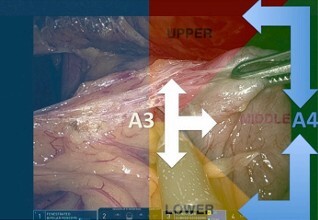
In this scenario, the arm closest to the optics (A3=Arm 3) changes position by running medially, and the most lateral arm (A4=Arm 4) changes position by rotating externally.

 The rules for proper arm movement can be seen in Figure 3. 

 Key "K" rules to improve robotic surgery skills:

1. Each quadrant can be occupied by one arm at a time.
2. Vacate the quadrant you want to occupy before activating the next arm.
3. The arm closest to the optic changes position running medially.
4. The most lateral arm (1 or 4) changes position by rotating externally.

## RESULTS

 Just like the "K" technique, activities in a dry-lab environment proved to be more capable of promoting the development of technical skills than training in VR^13,15^. This training model encourages facing more realistic work situations with the robotic platform. 

 One of the limiting factors for dry-lab training is the availability of dedicated robotic platforms for training, in addition to the cost of using training arms and the need for the presence of a qualified instructor. 

 Didactically, four main rules were established for using the K technique (Video 1 — link). 

**Video 1:**
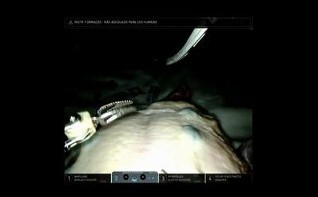
Standardization of robotic maneuvers using the K rules (link to the video)

 Rule number one allows for adequate work ergonomics, indicating which region should be occupied by each arm, whether active or static. When the arms occupy the same quadrant, there is a conflict of functions that impair performance and leads to a greater probability of collision between the effector parts of the arms. 

 A frequently made error occurs when changing arms, when the surgeon abandons the active arm to activate his auxiliary arm with the aim of occupying the same quadrant. This attitude generally leads to a limitation of the working space of the activated arm and requires a new change of arms to remove the inactive arm from the quadrant in question. Rule number two avoids this mistake. 

 Rules 3 and 4 prevent gripper arm collisions. These parts are often outside the field of vision and are therefore less easily diagnosed and less responsible for damage to the instrument or the patient. 

 The most common errors and examples of the applicability of the "K" technique in routine surgery can be seen in Video 2 — link. 

**Video 2:**
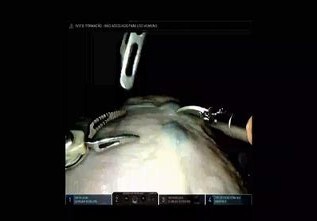
Standardization of robotic maneuvers using the K rules (link to the video)

## DISCUSSION

 Technical reports are very useful in transmitting learning and training in new technologies^4,8,9,12,15^. In the early 1990s, the development of practical skills in videolaparoscopy was fundamental for the dissemination of the technique. The success and reduction of the learning curve for those who learn the technique today are due to the standardization of training. Even performing a simple suture was the reason for publications on the standardization of movements^17^. 

 The da Vinci Technology Training Pathway is purely webbased, with limited access to hands-on training. The Brazilian Medical Association has regulated technical prerequisites for training in robotic surgery^1,8^. However, just like training and residency programs, the need for continued training and validation of learning does not have a well-defined standard^7^. Much of the training is limited to VR for reasons of practicality and cost. VR training has already demonstrated its effectiveness and safety. However, there is no consensus on which tasks and metrics are the most effective for the da Vinci Skills Simulators. The literature lacks well-designed studies^10,13-16^. 

 The exercise proposed by the "K" technique stimulates the development of five of the six domains defined by the Global Evaluative Assessment of Robotic Skills (GEARS)^5^ metrics: depth perception, bimanual dexterity, efficiency, autonomy, and robotic control. This exercise represents a challenge observed by everyone who transitions to robotic technique and can be practiced either on real training models or guide the construction of VR exercises to acquire the proposed skills^13^. 

## CONCLUSIONS

 The "K" technique is a relevant technical resource in the teaching of robotic surgery, as it provides an opportunity for the development of important skills for safety and optimization of surgical time. It is easily reproducible and can serve as a basis for building VR training models that explore its concepts at an even more affordable cost for a large scale of training. 

## Data Availability

The datasets generated and/or analyzed during the current study are available from the corresponding author upon reasonable request.

## References

[1] Araujo RLC, Benevenuto DS, Zilberstein B, Sallum RA, Aguiar S, Cavazzola LT (2020). Overview and perspectives about the robotic surgical certification process in Brazil: the new statement and a national web-survey. Rev Col Bras Cir.

[2] Claus C, Cavazzola L, Malcher F (2021). SubCutaneous OnLay endoscopic Approach (SCOLA) for midline ventral hernias associated with diastasis recti. Hernia.

[3] Costa TN, Tustumi F, Ferros LSM, Colonno BB, Abdalla RZ, Ribeiro U (2023). Robotic-assisted versus laparoscopic incisional hernia repair: differences in direct costs from a Brazilian Public Institute perspective. Arq Bras Cir Dig.

[4] Dubin AK, Smith R, Julian D, Tanaka A, Mattingly P (2017). A comparison of robotic simulation performance on basic virtual reality skills: simulator subjective versus objective assessment tools. J Minim Invasive Gynecol.

[5] Goh AC, Goldfarb DW, Sander JC, Miles BJ, Dunkin BJ (2012). Global evaluative assessment of robotic skills: validation of a clinical assessment tool to measure robotic surgical skills. J Urol.

[6] Guthart G (2023). Intuitive J.P. Morgan Healthcare Conference 2023 [Internet].

[7] Harji D, Houston F, Burke J, Griffiths B, Tilney H, Miskovic D (2023). The current status of robotic colorectal surgery training programmes. J Robot Surg.

[8] Leijte E, De Blaauw I, Rosman C, Botden SMBI (2024). Transferability of the robot assisted and laparoscopic suturing learning curves. J Robot Surg.

[9] Lima DL, Pinto RD, Trauczynski P, Liu J, Cavazzola LT (2024). Feasibility of image inversion for ventral hernia repair using the versius system. J Laparoendosc Adv Surg Tech A.

[10] Moglia A, Ferrari V, Morelli L, Ferrari M, Mosca F, Cuschieri A (2016). A systematic review of virtual reality simulators for robot-assisted surgery. Eur Urol.

[11] Nacul MP, Melani AGF, Zilberstein B, Benevenuto DS, Cavazzola LT, Araujo RLC (2020). Educational note: teaching and training in robotic surgery. An opinion of the Minimally Invasive and Robotic Surgery Committee of the Brazilian College of Surgeons. Rev Col Bras Cir.

[12] Rahimi AO, Ho K, Chang M, Gasper D, Ashouri Y, Dearmon-Moore D (2023). A systematic review of robotic surgery curricula using a contemporary educational framework. Surg Endosc.

[13] Raison N, Gavazzi A, Abe T, Ahmed K, Dasgupta P (2020). Virtually competent: a comparative analysis of virtual reality and dry-lab robotic simulation training. J Endourol.

[14] Raison N, Harrison P, Abe T, Aydin A, Ahmed K, Dasgupta P (2021). Procedural virtual reality simulation training for robotic surgery: a randomised controlled trial. Surg Endosc.

[15] Rapoport LM, Bezrukov EA, Tsarichenko DG, Martirosyan GA, Sukhanov RB, Krupinov GE (2019). Methods for training of robot-assisted radical prostatectomy. Khirurgiia (Mosk).

[16] Soper NJ, Hunter JG (1992). Suturing and knot tying in laparoscopy. Surg Clin North Am.

[17] Whittaker G, Aydin A, Raison N, Kum F, Challacombe B, Khan MS (2016). Validation of the RobotiX mentor robotic surgery simulator. J Endourol.

